# Air pollution and mortality in acute lymphoblastic leukemia patients across different age groups in Northern Thailand (1999–2020)

**DOI:** 10.1371/journal.pone.0355856

**Published:** 2026-08-13

**Authors:** Patrinee Traisathit, Lalita Sathitsamitphong, Thanawat Rattanathammethee, Pimwarat Srikummoon, Suttipong Kawilapat, Natthapat Thongsak, Nawapon Nakharutai, Salinee Thumronglaohapun, Titaporn Supasri, Phonpat Hemwan, Imjai Chitapanarux

**Affiliations:** 1 Department of Statistics, Faculty of Science, Chiang Mai University, Chiang Mai, Thailand; 2 Data Science Research Center, Department of Statistics, Faculty of Science, Chiang Mai University, Chiang Mai, Thailand; 3 Department of Pediatrics, Faculty of Medicine, Chiang Mai University, Chiang Mai, Thailand; 4 Department of Internal Medicine, Faculty of Medicine, Chiang Mai University, Chiang Mai, Thailand; 5 Atmospheric Research Unit of National Astronomical Research Institute of Thailand, Chiang Mai, Thailand; 6 Department of Geography, Faculty of Social Sciences, Chiang Mai University, Chiang Mai, Thailand; 7 Northern Thai Research Group of Therapeutic Radiology and Oncology (NTRG-TRO), Division of Radiation Oncology, Faculty of Medicine, Chiang Mai University, Chiang Mai, Thailand; Tsinghua University, CHINA

## Abstract

This study investigated the impact of air pollution on 604 patients diagnosed with acute lymphoblastic leukemia (ALL) between 1999 and 2020 in upper northern Thailand. Patients were categorized into three age groups: pediatric (<15 years), adolescent and young adult (AYA) (15–39 years), and adult (≥40 years). The cohort included 344 (57.0%) pediatric, 152 (25.1%) AYA, and 108 (17.9%) adult patients, with 5-year survival rates of 64.5%, 19.6%, and 6.7%, respectively. Concentrations of PM_2.5_, PM_10_, O₃, and CO were significantly higher in patients who died, particularly among pediatric cases. Among AYA patients, those who died had higher levels of PM_2.5_, PM_10_, and CO than survivors. Compared to the pediatric group, the risk of death was higher in AYA (adjusted hazard ratio [aHR]: 3.76) and adult (aHR: 8.10) groups. Patients diagnosed before 2010 had an increased risk of death (aHR: 1.38). Notably, PM_2.5_ levels ≥50 µg/m³ were associated with increased mortality in pediatric patients. These findings highlight the adverse effects of air pollution on survival outcomes in ALL patients, particularly children under 15 years of age.

## Introduction

Acute lymphoblastic leukemia (ALL), the most common cancer in children [1], is approximately four times higher in children than in adults [2], with the highest incidence occurring from 2 to 10 years of age [3]. Data from the Surveillance, Epidemiology, and End Results (SEER) program have shown significant variation in 5-year survival rates for ALL patients by age. Pediatric patients (<15 years) have the highest 5-year survival rate (85%), followed by those aged 15–19 years old (61%), 30–39 years old (40%), and 40 years and older (< 30%) [4]. Multicenter studies in Thailand have demonstrated similar trends of overall survival (OS) and event-free survival (EFS) among pediatric patients (< 15 years old), adolescents and young adults (AYA; 15–39 years old), and adults (≥ 40 years old). The 5-year OS and EFS have been reported as 75% and 81.7%, respectively, for ALL pediatric patients [5], while the 2-year OS and EFS are lower for AYA (54% and 45.5%, respectively) and the adult populations (44.1% and 26.2%, respectively) [6]. However, advancements in treatment modalities over time have led to a significantly improved ALL survival rate for all age groups since 2010, increasing from 51% before 1990 to 72% afterward [4]. Nevertheless, the prognosis remains poor, particularly for adults (≥ 40 years old) [1].

Air pollution is one of the most significant challenges of our time as it detrimentally affects health, thereby contributing to higher rates of illness and death [7–9]. In northern Thailand, air pollution, particularly from airborne particulate matter (PM) due to biomass burning, is especially severe from January to April each year. In particular, this issue is exacerbated by smoke from burning activities in neighboring countries [10,11]. The World Health Organization (WHO) estimates that approximately 7 million deaths, predominantly from noncommunicable diseases, are linked to the combined impact of ambient and household air pollution [12]. Regarding the WHO’s 2021 guidelines, the 24-hour average concentration of PM_2.5_ should not exceed 15 µg/m^3^ [12]. However, data from northern Thailand in 2020 revealed that PM_2.5_ concentrations ranged from 16 to 195 µg/m^3^ [11], greatly exceeding the levels recommended by the WHO. Therefore, air pollution poses a serious health concern, and effective pollution management is needed to protect public health.

Over the past few years, numerous studies have highlighted the negative health impacts associated with both short-term and long-term exposure to air pollutants [8]. In particular, exposure to various air pollutants, including PM, nitrogen dioxide (NO_2_), ozone (O_3_), and carbon monoxide (CO), has been linked with increased mortality and constitutes a significant health risk [7,8,13]. Epidemiological evidence on the impact of outdoor air pollution on cancers other than lung cancer is relatively sparse, highlighting the need to understand its effects on cancer incidence and survival across other cancer types [14]. An increase in PM_2.5_ concentration by 5 µg/m^3^ has been associated with mortality in young children with lymphoid leukemia [5-year hazard ratio (HR_5-year_): 1.32; 95% confidence interval (CI): 1.02–1.71]. Nevertheless, no significant association between an increase in PM_2.5_ concentration and leukemia mortality in AYA has been demonstrated [15]. However, evidence on the impact of ambient air pollution on the survival of patients with ALL, particularly across age groups in regions with extreme exposure, remains limited. The aim of the present study is to address this knowledge gap by investigating whether PM_2.5_, PM_10_, NO_2_, O_3_, and CO exposure is associated with the mortality rates of pediatric, AYA, and adult patients diagnosed with ALL in eight provinces in upper northern Thailand.

## Materials and methods

### Study design and population

This was a retrospective study involving a cohort of 604 pediatric patients (< 15 years old), AYA (15-39 years old), and adults (≥ 40 years old) in eight provinces in upper northern region of Thailand: Chiang Mai, Chiang Rai, Nan, Phrae, Phayao, Lampang, Lamphun, and Mae Hong Son diagnosed with ALL between January 1, 1999 and December 31, 2020. The patients were tracked from their registration date until the end of February 2024 to assess their survival rates. We access this clinical data on May 1, 2024.

### Data collection and measurements

The cancer characteristics and stage at diagnosis, all demographic data available in the Chiang Mai Cancer Registry database (such as sex, age, body mass index (BMI), smoking history, hill tribe ethnicity, and a family history of cancer) were included in the analysis to identify any potential risk factors. For the pediatric patients, additional demographic data (such as maternal smoking history, age at delivery and parity, and the family’s educational background) were also included. Cause of death was obtained from the Chiang Mai Cancer Registry based on linked hospital records and national death certificate data.

Data on the hourly concentrations of PM_2.5_, PM_10_, NO_2_, O_3_, and CO were obtained from the Copernicus Atmosphere Monitoring Service at the European Centre for Medium-Range Weather Forecasts (ECMWF) [16,17]. This dataset comprising the most recent global reanalysis of atmospheric composition includes consistent 3D fields of various aerosols and chemical species with a horizontal resolution of approximately 80 km (often interpolated to a 0.75° * 0.75° grid). Using this data, we calculated the annual average concentrations of PM_2.5_ (µg/m³), PM_10_ (µg/m³), NO_2_ (ppb), O_3_ (ppb), and CO (ppb) for 2003–2020 for each of the districts in the eight provinces in upper northern Thailand. These averages were then linked to the corresponding districts for the addresses of 604 patients provided in the Chiang Mai Cancer Registry dataset at the year of diagnosis. We assumed that the patients’ recorded addresses did not change during the study period. The pollution data were updated annually until the patient’s death, loss to follow-up, or data censoring. Since residential mobility during follow-up could not be assessed, exposure misclassification is possible if patients relocated after diagnosis, thereby potentially attenuating the observed associations.

ALL cases had been histologically confirmed (ICD 10: C91.0) and recorded in the Chiang Mai Cancer Registry. Patients were excluded if they had secondary or therapy-related leukemia, missing or unverifiable residential district information at the time of diagnosis (precluding exposure assignment), resided outside of the eight provinces included in the study, had an unknown vital status, or had incomplete follow-up data. Patients diagnosed before 2003 were retained. For the included cases, air pollution exposure was assigned beginning in 2003 (when CAMS data became available). We have also clarified that missing covariate data were handled via a complete case analysis of each model.

### Ethical considerations

The study was conducted in accordance with the Declaration of Helsinki and approved by the Ethics Committee of the Faculty of Medicine, Chiang Mai University (No. 200/2021). Patient consent was waived due to anonymous data recorded in the present study.

### Statistical analysis

The baseline characteristics of the study participants are described using medians and interquartile ranges (IQRs) for continuous variables and frequencies and percentages for categorical variables. The follow-up period was measured from the date of diagnosis to either the date of death from any cause or the last follow-up date, whichever occurred first. The annually averaged concentrations of PM_2.5_, PM_10_, NO_2_, O_3_, and CO were segmented into two categories using quartiles, to which dichotomization was applied when deemed suitable.

The Shapiro–Wilk test was utilized to assess the normality of continuous variables including demographics and pollutant concentrations. For data exhibiting normal distributions, parametric tests were employed, such as the independent t-test or one-way analysis of variance for comparing mean concentrations between groups. Conversely, non-normally distributed data were analyzed using non-parametric tests, including the Mann-Whitney U test or Kruskal-Wallis test for comparing median concentrations between groups.

The overall mortality rate and the rates associated with each variable were determined by dividing the number of deaths by the total person-years of follow-up. The date used for survival analysis was the date of diagnosis, as recorded in the cancer registry database. Kaplan-Meier curves were used to estimate survival rates, and the significance of the differences in survival probabilities between groups for each variable was assessed using log-rank tests. Cox proportional hazard models were used to investigate the associations between the risk of death in ALL patients and various risk factors, including gender, age, hill tribe ethnicity, year of diagnosis, a family history of cancer, smoking history, and time-updated concentrations of PM_2.5_, PM_10_, NO_2_, O_3_, and CO. Variables with *p* < 0.25 in the univariable analysis were included in the multivariable model [18]. All statistical analyses were performed using Stata (version 18).

## Results

### Characteristics of the ALL patients

Of the 604 patients with ALL registered between 1999 and 2020, 344 (57.0%) were pediatric, 152 (25.1%) were AYA, and 108 (17.9%) were adults. The median age at diagnosis was 11.7 years old (IQR: 4.6–30.7), with 55.8% of the patients being male. The median ages of the pediatric, AYA, and adult patient groups were 5.1 years old (IQR: 3.0–8.5), 22.3 years old (IQR: 17.6–31.3), and 55.6 years old (IQR: 47.7–63.9), respectively (Table 1).

**Table 1:** Characteristics of the study population by age group.

Characteristic	Total (N = 604)	Pediatric (N = 344)	AYA (N = 152)	Adult (N = 108)
Age (years old), median (IQR)	11.7 (4.6-30.7)	5.1 (3.0-8.5)	22.3 (17.6-31.3)	55.6 (47.7-63.9)
Gender: male, *n* (%)	337 (55.8)	197 (57.3)	87 (57.2)	53 (49.1)
BMI (kg/m^2^), median (IQR)	16.9 (14.7-20.7)	15.2 (14.0-17.1)	20.5 (18.4-24.2)	22.4 (20.2-24.8)
Hill tribe ethnicity, *n* (%)	86 (14.2)	57 (16.6)	24 (15.8)	5 (4.6)
Province, *n* (%)				
Chiang Mai	294 (48.7)	169 (49.1)	69 (45.4)	56 (51.9)
Chiang Rai	64 (10.6)	36 (10.5)	19 (12.5)	9 (8.3)
Lamphun	55 (9.1)	30 (8.7)	14 (9.2)	11 (10.2)
Phayao	49 (8.1)	30 (8.7)	10 (6.6)	9 (8.3)
Mae Hong Son	43 (7.1)	29 (8.4)	11 (7.2)	3 (2.8)
Nan	37 (6.1)	22 (6.4)	5 (3.3)	10 (9.3)
Lampang	32 (5.3)	11 (3.2)	15 (9.9)	6 (5.6)
Phrae	30 (5.0)	17 (4.9)	9 (5.9)	4 (3.7)
Family history of cancer, *n* (%)	26 (4.3)	12 (3.5)	8 (5.3)	6 (5.6)
Maternal Smoking, *n* (%)		29 (8.4)		
Smoking history, *n* (%)			21 (13.8)	17 (15.7)
Year of diagnosis, *n* (%)				
1999-2009	337 (55.8%)	198 (57.6%)	84 (55.3%)	55 (50.9%)
2010-2020	267 (44.2%)	146 (42.4%)	68 (44.7%)	53 (49.1%)
Dead, *n* (%)	331 (54.8)	126 (36.6)	108 (71.1)	97 (89.8)
Cause of death, *n* (%)				
Cancer-related dead	311 (94.0)	111 (88.1)	105 (97.2)	95 (97.9)
Others	20 (6.0)	15 (11.9)	3 (2.8)	2 (2.1)

AYA, adolescent and young adult; IQR, interquartile range; BMI, body mass index; N, number of patients in the subgroup; n, number of patients in each category. Maternal smoking was recorded for pediatric patients only. Smoking history was recorded for AYA and adult patients only. Percentages were calculated using the relevant subgroup denominator.

### Survival rates

**Fig 1:**
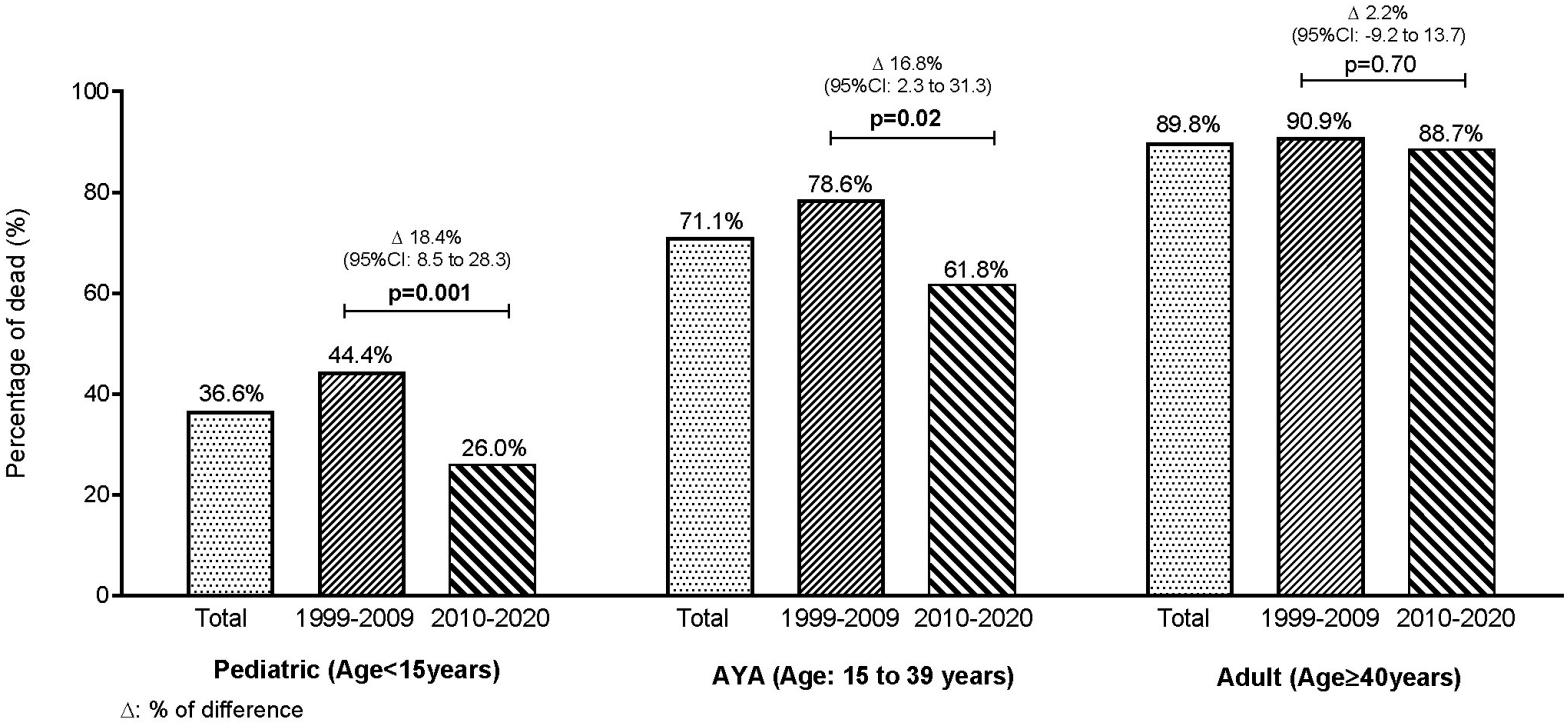
Bar charts showing percentages of deceased acute lymphoblastic leukemia patients by age group and time period of diagnosis.

The highest proportion of deaths occurred among the adult group (89.8%), followed by the AYA group (71.1%) and pediatric group (36.6%). For the pediatric group, the percentage of deaths decreased significantly from 44.4% in those diagnosed before 2010 to 26.0% in those diagnosed since 2010, representing a reduction of 18.4% (95% CI: 8.5–28.3; *p* = 0.001). A similar trend was seen in the AYA group (from 78.6% in those diagnosed before 2010 to 61.2% in those diagnosed since 2010), signifying a reduction of 11.6% (95% CI: 2.2–31.3; *p* = 0.02). However, the decrease was only slight in the adult group (from 90.9% in those diagnosed before 2010 to 82.7% in those diagnosed since 2010); this reduction of 4.2% (95% CI: −2.0 to 10.3; *p* = 0.07) was not statistically significant (Fig 1).

**Fig 2:**
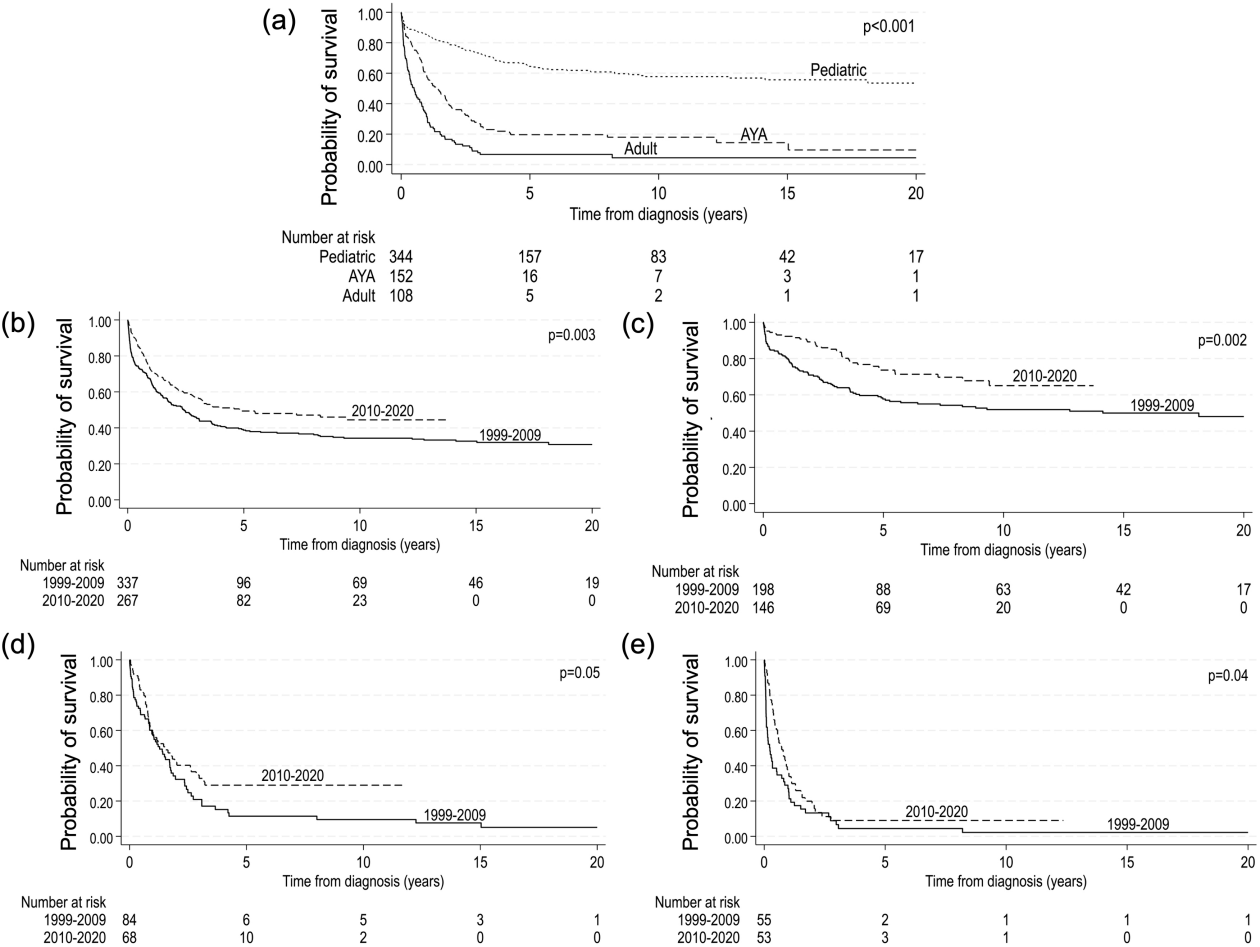
The survival rates of participants diagnosed with acute lymphoblastic leukemia categorized by (a) age group, (pediatric (age < 15 years old), AYA (adolescent and young adult; age 15–39 years old), and adult (age ≥ 40 years old)) and stratified by year of diagnosis (1999–2009 or 2010–2020): (c) the pediatric group, (d) the AYA group, and (e) the adult group.

The survival rate for the adult group was poorer than those for the pediatric and AYA groups (*p* < 0.001) (Fig 2(a)), which highlights the impact of age on survival time. Moreover, the survival probability of the ALL patients improved when diagnosed since 2010 compared to those diagnosed beforehand (*p* = 0.003) (Fig 2(b)). Fig 2(c) illustrates that pediatric patients diagnosed since 2010 had a significantly better survival rate compared to those diagnosed beforehand (*p* = 0.002). Similarly, AYA patients diagnosed since 2010 showed an improved survival probability compared to those diagnosed beforehand, albeit that the statistical significance is borderline (*p* = 0.05) (Fig 2(d)). For adults diagnosed since 2010, the survival rate was slightly better than those diagnosed beforehand, which was also statistically significant (*p* = 0.04) (Fig 2(e)).

The results in Table 2 indicate that the overall median follow-up time for the cohort was 1.9 years (IQR: 0.5–6.4) and, specifically, 4.3 years (IQR: 1.2–9.5), 1.0 years (IQR: 0.4–2.4), and 0.4 years (IQR: 0.1–1.1) for the pediatric, AYA, and adult groups, respectively. The overall 5-year survival rate was 43.4%, with a range from 39.2% to 47.6%, reflecting a median survival time of 3.1 years. The 5-year survival rates were 64.5% for the pediatric group, 19.6% for the AYA group, and 6.7% for the adult group. Survival rates stratified by the time of diagnosis indicate that the 5-year survival rate increased from 38.8% for those diagnosed before 2010 to 49.4% for those diagnosed since 2010, and the median survival time was extended by 4.8 years. The 5-year survival rate for the adult group was the least favorable at 6.7% (95% CI: 2.8%−12.9%) with a median survival time of 0.5 years. When stratified by the time of diagnosis, adults diagnosed before 2010 had a survival rate of 4.4% (95% CI: 0.8%−13.1%), improving to 9.0% (95% CI: 3.0%−19.1%) for those diagnosed since 2010.

**Table 2:** The 5-year overall survival rate by age group and year of diagnosis.

Year of diagnosis	Median Follow-Up Time (y (IQR))	Five-Year Overall Survival Rate (%)	95% CI (%)		Median Survival Time (y)	*p*
	**Lower**	**Upper**	
**Total**	**1.9 (0.5-6.4)**	**43.4**	**39.2**	**47.6**	**3.1**	
1999-2009	1.5 (0.3-6.8)	38.8	33.2	44.3	2.4	0.003
2010-2020	2.4 (0.7-6.3)	49.4	42.8	55.6	4.8	
**Pediatric (Age < 15 years old)**	**4.3 (1.2-9.5)**	**64.5**	**58.8**	**69.6**	**2.5**	
1999-2009	3.8 (1.0-13.8)	57.7	50.1	64.6	14.1	0.002
2010-2020	4.7 (2.2-8.1)	73.7	64.8	80.6	NR^a^	
**AYA (Age: 15–39 years old)**	**1.0 (0.4-2.4)**	**19.6**	**12.8**	**27.4**	**1.4**	
1999-2009	1.1 (0.3-1.9)	11.4	4.8	21.2	1.3	0.05
2010-2020	0.9 (0.5-3.1)	29.0	17.8	41.0	1.5	
**Adult (Age ≥ 40 years old)**	**0.4 (0.1-1.1)**	**6.7**	**2.8**	**12.9**	**0.5**	
1999-2009	0.2 (0.1-1.0)	4.4	0.8	13.1	0.2	0.04
2010-2020	0.7 (0.3-1.3)	9.0	3.0	19.1	0.7	

AYA, adolescent and young adult; IQR, interquartile range; CI, confidence interval.

NR^a^, not reached, meaning that the median survival time of pediatric patients diagnosed since 2010 was unavailable due to the survival rate not reaching the 50% probability of survival during the study period.

### Pollutant concentrations

The median annual concentrations of the pollutants, as depicted in S1 Fig, suggest a temporal decrease in levels for PM_2.5_, PM_10_, and CO. Given the non-normal distribution of pollutant concentrations (S1 Table), median comparisons between patients who survived and died, as well as across years of diagnosis were presented in S2 Table. The median concentrations of PM_2.5_, PM_10_, NO_2_, O_3_, and CO were 35.8 µg/m³, 49.8 µg/m³, 7.2 ppb, 36.4 ppb, and 378.9 ppb, respectively. A detailed comparison of median concentrations for each pollutant across age groups and years of diagnosis within those age group is presented in S2 Fig. This comparison reveals no significant differences among the concentrations of most of the pollutants (PM_2.5_, PM_10_, O_3_, and CO) across the age groups, suggesting consistent exposure levels irrespective of age. However, an exception was noted for NO_2_ where the adult group exhibited the highest median concentration at 6.8 ppb, followed by AYA at 7.9 ppb and pediatric at 8.2 ppb (p < 0.001) (S2 Fig. (e)).

According to S2 Table, concentrations of PM_2.5_ (*p* < 0.001), PM_10_ (*p* < 0.001), O_3_ (*p* = 0.007), and CO (*p* < 0.001) were significantly higher in the patients who had died. Among pediatrics, PM_2.5_ (*p* < 0.001), PM_10_ (*p* < 0.001), O_3_ (*p* < 0.001), and CO (*p* < 0.001) concentrations were also significantly higher in those who had died. For the AYA group, PM_2.5_ (*p* = 0.001), PM_10_ (*p* = 0.001), and CO (*p* < 0.001) concentrations were significantly higher in the patients who had died compared to those who survived. Conversely, in the adult group, there were no significant differences in the median of PM_2.5_, PM_10_, NO_2_, and O_3_ concentrations between those who had died and those who survived, except for CO (*p* = 0.036). Further analysis, presented in S2 Table and S2 Fig, compares the median annual pollutant concentrations in the residential districts of the patients stratified by years of diagnosis (1999–2009 vs. 2010–2020) for each age group. It was found that the concentrations of all pollutants were significantly lower in the later period across all age groups, with the exception of O_3_ concentration in the adult group (*p* = 0.309).

### Death risk factors

The results of the univariable and multivariable analyses for determining the risk factors for death in ALL patients are provided in Table 3. The results of the univariable analysis indicate that age, specifically the AYA and adult groups, posed the highest risk factors for death (*p* < 0.001). The results of the multivariable analysis further demonstrate that being in the AYA group (aHR: 3.76, 95% CI: 2.77–5.10) or the adult group (aHR: 8.10, 95% CI: 5.85–11.23), or being diagnosed with ALL before 2010 (aHR: 1.38, 95% CI: 1.07–1.78), were independently associated with an increased risk of death. Subgroup analysis by age group reveals that the pediatric patients exposed to higher concentrations of PM_2.5_ (≥ 50 µg/m^3^) had an increased risk of death (HR: 2.02; 95% CI: 1.10–3.70) in the univariable analysis (S3 Table). However, this association was not statistically significant in the multivariable analysis (aHR: 1.92; 95% CI: 0.96–3.83). Despite this, ALL diagnosis before 2010 still posed a higher risk of death in the pediatric group (aHR: 1.72; 95% CI: 1.06–2.80) (S3 Table). For the AYA and adult groups, no significant links were found between the pollutants and death.

**Table 3:** Risk factors associated with death among patients with acute lymphoblastic leukemia.

Factors	Univariable Analysis			Multivariable Analysis		
	HR	95% CI	*p*	aHR	95% CI	*p*
**At diagnosis**						
**Age group**						
Pediatric	Ref.	–	–	–	–	–
AYA	3.44	2.64-4.48	<0.001	3.76	2.77-5.10	<0.001
Adult	6.74	5.10-8.91	<0.001	8.10	5.85-11.23	<0.001
Male	1.01	0.82-1.26	0.90	–	–	–
Hill tribe	0.78	0.54-1.11	0.16	1.28	0.87-1.89	0.21
**Year of diagnosis**						
1999-2009	1.40	1.12-1.75	0.003	1.38	1.07-1.78	0.01
2010-2020	Ref.	–	–	–	–	–
Family history of cancer	0.99	0.60-1.64	0.97	–	–	–
Smoking status (yes vs. no)^a^	1.10	0.79-1.54	0.57	–	–	–
**Time-updated variables**						
PM_2.5_ concentration ≥ 50 µg/m^3^	1.40	0.91-2.14	0.13	1.38	0.89-2.13	0.15
PM_10_ concentration ≥ 55 µg/m^3^	1.02	0.78-1.33	0.91	–	–	–
NO_2_ concentration ≥ 8.5 ppb	1.01	0.77-1.33	0.95	–	–	–
O_3_ concentration ≥ 37.8 ppb	0.94	0.71-1.25	0.68	–	–	–
CO concentration ≥ 410 ppb	1.06	0.82-1.38	0.66	–	–	–

HR, hazard ratio; aHR, adjusted hazard ratio; CI, confidence interval; Ref., reference group; AYA, adolescent and young adult; PM_2.5_, particulate matter < 2.5 μm; PM_10_, particulate matter < 10 μm; NO_2_, nitrogen dioxide; O_3_, ozone; CO, carbon monoxide.

^a^Smoking status: maternal smoking for the pediatric group and smoking history for the AYA and adult groups.

However, stratifying the AYA patients into those aged 30–39 and 15–19 years old revealed a higher risk of death in the former (aHR: 1.69; 95% CI: 1.01–2.85) (S4 Table). Similarly, stratifying the adult group into those aged ≥ 60 and 40–49 years old disclosed a higher risk of death in the former (aHR: 1.97; 95% CI: 1.21–3.19) (S5 Table).

## Discussion

This retrospective cohort study evaluated survival and explored associations between ambient air pollution and mortality among 604 patients diagnosed with ALL in upper northern Thailand between 1999 and 2020. The cohort was stratified into pediatric (< 15 years old), adolescent and young adult (AYA; 15–39 years old), and adult (≥ 40 years old) groups. Survival differed strongly by age group, with the highest five-year survival rate in the pediatric group and substantially lower survival rates in the older groups. In the multivariable analyses, mortality risk was markedly higher in AYA and adult patients compared with pediatric patients (aHR values of 3.76 and 8.10, respectively), thereby highlighting the predominant role of age-related and clinical factors in ALL survival.

Crude comparisons suggest that patients who died had been exposed to higher ambient concentrations of PM_2.5_, PM_10_, O_3_, and CO, which was particularly prevalent in the pediatric group. However, when applying the adjusted model, evidence for an independent association between air pollution and mortality was limited. Pediatric ALL patients exposed to PM_2.5_ ≥ 50 µg/m³ showed an increased hazard estimate (aHR: 1.92; 95% CI 0.96–3.83), albeit this finding did not quite reach statistical significance (*p* = 0.06). Nevertheless, this trend suggests that monitoring the effect of PM_2.5_ on pediatric ALL patients should be further investigated.

Regarding the survival rates across the two diagnostic periods (1999–2009 vs. 2010–2020), those diagnosed with ALL during the latter period had a lower mortality risk compared to those diagnosed in the former period. This improvement was statistically significant for the pediatric and adult ALL populations, as was that for the AYA population, albeit with borderline significance in the latter case. The difference in survival rates between the two diagnostic periods is likely due to improved healthcare access, treatment protocols [5,6,19,20], and supportive care measures implemented over the latter period.

The Thai Pediatric Oncology Group (ThaiPOG) launched the first leukemia national protocol in 2006, with advances in treatment for pediatric cancers being subsequently established in 2014. The Thai Acute Leukemia Working Group, founded in 2012, established the national guidelines for adult patients with leukemia. These national protocols cover standard treatment guidelines, comprehensive supportive care, and ongoing protocol development for all patients with leukemia in Thailand, with the aim of improving treatment outcomes over time. A previous study on pediatric ALL treatment in Thailand found that the implementation of modified treatment protocols improved the overall outcomes for patients, albeit relapse was reported as still being challenging [5,19]. Adapting the pediatric ALL treatment regimen could provide a new treatment strategy for AYA patients with ALL [21], such as that reported for a single-center study in Thailand [20], with better EFS and OS for AYA who had received the pediatric-adapted protocol. However, despite the advances in care and treatment, the persistently high mortality rates among AYA and adult patients highlight the need for further analysis to determine appropriate treatment protocols and other treatment modalities.

While taking the establishment of an effective national treatment protocol into account, we hypothesized that exposure to air pollution might impact the survival outcomes of ALL patients. From January to May, PM_2.5_ levels in Lampang, Chiang Rai, and Chiang Mai provinces were notably high compared to the WHO guidelines, ranging from 150 to 195 µg/m^3^ [11]. Moreover, air pollution exposure is known to be a major risk factor influencing long-term public health, especially in the upper northern region of Thailand. The long study period (1999–2020) may have introduced temporal confounding due to changes in treatment, diagnostics, and pollution levels. Although we temporally divided the data into two diagnostic periods (before 2010 vs. 2010 onward), unmeasured time-varying factors might still have persisted. Notably, pediatric patients demonstrated a more pronounced sensitivity to elevated PM_2.5_ levels (aHR = 1.92; 95% CI = 0.96–3.83; *p* = 0.06), suggesting that they are particularly vulnerable to the adverse effects of air pollution. This is consistent with an analysis of data from the Utah Cancer Registry on the effect of PM_2.5_ exposure and mortality among pediatric cancer patients [15]; the authors found a significant association between increments of 5 µg/m^3^ in PM_2.5_ exposure and all-cause mortality in patients with lymphoid leukemia, lymphoma, central nervous system tumors, and hepatic tumors at 5 and 10 years post diagnosis. Thus, air pollution avoidance and effective pollution management policy should be implemented to protect vulnerable populations from air pollution exposure.

Exposure to air pollution reportedly activates systemic inflammation, increases oxidative stress [22–26], and disturbs the immune system [27], which could affect treatment responses and survival outcomes of cancer patients. Specifically, an association between PM_2.5_ concentration and inflammatory markers was reported by Hoffmann et al. [28]. This suggests that persistent inflammatory activation due to prolonged exposure to air pollution could influence the survival outcomes of ALL patients. In the present study, we found that pediatric ALL patients had a longer follow-up time and lower mortality rate than the AYA and adult patients. Hence, we postulate that pediatric ALL patients were exposed to a greater level of air pollution due to the longer follow-up period, which could have impacted their health and longevity [15]. However, since data on inflammatory markers were absent for our cohort, a future study is required to better understand the effects of inflammation and oxidative stress on ALL progression.

Although there were 4- and 8-fold higher risks of death in the AYA and adult groups compared to the pediatric group, respectively, air pollution did not increase this risk in the former two cases. The findings from subgroup analyses of the AYA group (30–39 vs. 15–19 years old) and the adult group (60 vs. 40–49 years old) suggest that being older is significantly associated with a higher risk of death. This is in agreement with the findings of [29], who reported that older age is associated with an increased risk of treatment-related toxicity and a worse survival rate compared to younger adults. This is likely because treating ALL in older adults is challenging due to the aggressive biology of the disease, the presence of comorbidities, poor treatment tolerance, and a poor response to conventional chemotherapy [1,30,31]. Furthermore, the limited follow-up data used in our study restricts the precision and stability of long-term survival estimates for the adult group and could have reduced the ability to detect an association between air pollution exposure and mortality in this group. We further note that the sparse long-term follow-up data for the adult group likely reflects the poor prognosis and rapid disease progression in this population, which necessitates cautious interpretation of the results. We recommend that future studies with longer follow-up times and more detailed air pollution exposure assessments are needed to better evaluate the influence of air pollution on adult ALL survival.

The observed association between air pollution and ALL mortality underscores the importance of integrating environmental health considerations into oncologic care. Healthcare providers in regions with high pollution levels should consider implementing protective measures, such as air filtration systems and advisories on outdoor activities during periods of elevated pollution. Furthermore, public health initiatives aimed at reducing emissions and mitigating air pollution could have downstream benefits in improving cancer survival outcomes. Future research should focus on longitudinal studies with individual-level pollution exposure assessments to better elucidate the mechanisms linking air pollution to ALL outcomes. In addition, investigating gene-environment interactions may provide deeper insights into why certain subpopulations, such as pediatric patients, exhibit heightened vulnerability. Expanding the study to include other forms of leukemia and malignancies could further clarify the broader implications of air pollution on cancer prognosis.

A further consideration is the relatively short mean follow-up times overall and particularly for the adults (1.9 and 0.4 years, respectively). While we estimated five-year survival using standard survival methods, longer-term estimates for groups with limited follow-up should be interpreted as less stable. Future studies with longer follow-up and richer clinical data are essential to better characterize long-term survival patterns and the role of pollution exposure.

Major strengths of this study are the long assessment period, the large cohort size, and the comprehensive dataset, which allowed for stratification by age and diagnostic period. In addition, the incorporation of real-time air pollution data provided valuable insights into exposure experienced by the study population. However, several limitations must be acknowledged. First, the observational nature of the study precludes definitive causal inferences. Second, reliance on pollution data at the district level rather than the individual level could have introduced exposure misclassification bias. A longitudinal study using individual-level pollution assessments and investigating gene-environment interactions may provide better results for a more precise interpretation. Third, the high proportion of missing data regarding maternal age, education level, and smoking history could have potentially biased the results. Utilizing the model with complete data might help to eliminate these sources of bias. The improved survival rate over time is most plausibly driven by treatment and healthcare improvements. Despite the association that PM_2.5_ exposure increases ALL mortality found in this study, this is an unproven contributor and should be further investigated. Confounding by treatment protocol, disease severity, socioeconomic status, and healthcare access could have affected this study’s findings. However, data concerning these potentially confounding variables were unavailable in the dataset used in this study, so adjusting for them could not be carried out. To make interpretation and monitoring easier, the annually averaged concentrations of PM_2.5_, PM_10_, NO_2_, O_3_, and CO were segmented into two categories using quartiles, to which dichotomization was applied when deemed suitable. However, categorizing continuous pollutant concentrations using these cut-offs might have reduced the analytical precision and statistical power compared with continuous exposure–response modeling. Using continuous variables or other cut-offs in the analysis should be considered in a future study. Next, the follow-up period for survival outcomes was relatively short, particularly for the adult group, which may limit the robustness of the long-term survival estimates. Using patient residency to link with the area-level pollution data could have potentially introduced bias. A more precise assessment of air pollution exposure should be considered in a future study. Moreover, residential mobility during follow-up could not be assessed, exposure misclassification is possible if patients relocated after diagnosis, thereby potentially attenuating the observed associations. Multiple pollutants and subgroup analyses were conducted without formal adjustment for multiple comparisons. Our findings should be interpreted cautiously and considered as exploratory in nature. Additionally, further study should include other pollutants such as benzene from vehicular traffic, which has been found to be associated with leukemia [32]. Generalizability of our findings might be limited because this study reflects exposure patterns and clinical contexts in upper northern Thailand only. Expanding the investigation to other settings is warranted.

## Conclusions

Although the pollution concentrations were not statistically significantly associated with the mortality of ALL pediatric patients, the findings from this study suggest that air pollution, especially PM_2.5_, detrimentally affects health outcomes. In the AYA and adult groups, being older was found to be associated with a higher risk of death from ALL. Addressing environmental risk factors alongside advancements in medical treatment may provide a more holistic approach to improving survival rates in this vulnerable population.

## Supporting information

S1 Table Normality tests of continuous variables. (DOCX)

S2 Table The median concentrations of pollutants by age group according to survival status and year of diagnosis (1999–2009 or 2010–2020). (DOCX)

S3 Table Risk factors associated with death in the pediatric group with acute lymphoblastic leukemia. (DOCX)

S4 Table Risk factors associated with death in the adolescent and young adult group with acute lymphoblastic leukemia. (DOCX)

S5 Table Risk factors associated with death in the adult group with acute lymphoblastic leukemia. (DOCX)

S1 Fig Average annual concentrations of (a) PM_2.5_ (particulate matter < 2.5 μm), (b) PM_10_ (particulate matter < 10 μm), (c) NO_2_ (nitrogen dioxide), (d) O_3_ (ozone), and (e) CO (carbon monoxide). (DOCX)

S2 Fig The median concentrations of (a) PM_2.5_ by age group, (b) PM_2.5_ by age group and year of diagnosis, (c) PM_10_ by age group, (d) PM_10_ by age group and year of diagnosis, (e) NO_25_ by age group, (f) NO_2_ by age group and year of diagnosis, (g) O_3_ by age group, (h) O_3_ by age group and year of diagnosis, (i) CO by age group, (j) CO by age group and year of diagnosis during the diagnosis of ALL to death or last visit. SD, standard deviation; PM_2·5_, particulate matter < 2·5 μm; PM_10_, particulate matter < 10 μm; NO_2_, nitrogen dioxide; O_3_, ozone; CO, carbon monoxide.
(DOCX)
