# Supplementary material for: Air pollution and mortality in acute lymphoblastic leukemia patients across different age groups in Northern Thailand (1999–2020)
Source: PLoS One. 2026 Aug 13;21(8):e0355856. doi: 10.1371/journal.pone.0355856 (PMC13472421; doi:10.1371/journal.pone.0355856)
Supplement: Supplementary file 1 [file pone.0355856.s001.docx]

# **S1 Table.** **Normality tests of continuous variables.**

| **Variables** | **Observations** | **W-statistic** | **Z-statistic** | ***p*-value^a^** |
| --- | --- | --- | --- | --- |
| Age at diagnosis | 604 | 0.826 | 10.285 | <0.001 |
| Weight | 439 | 0.919 | 7.620 | <0.001 |
| Height | 402 | 0.933 | 6.961 | <0.001 |
| BMI | 402 | 0.903 | 7.821 | <0.001 |
| PM_2.5_ | 2,538 | 0.889 | 13.080 | <0.001 |
| PM_10_ | 2,538 | 0.893 | 12.979 | <0.001 |
| NO_2_ | 2,538 | 0.970 | 9.719 | <0.001 |
| O_3_ | 2,538 | 0.859 | 13.694 | <0.001 |
| CO | 2,538 | 0.968 | 9.845 | <0.001 |

BMI, body mass index; PM_2.5_, particulate matter < 2.5 μm; PM_10_, particulate matter < 10 μm; NO_2_, nitrogen dioxide; O_3_, ozone; CO, carbon monoxide.

^a^ *p*-values derived from the Shapiro-Wilk test
