# Supplementary material for: Air pollution and mortality in acute lymphoblastic leukemia patients across different age groups in Northern Thailand (1999–2020)
Source: PLoS One. 2026 Aug 13;21(8):e0355856. doi: 10.1371/journal.pone.0355856 (PMC13472421; doi:10.1371/journal.pone.0355856)
Supplement: Supplementary file 2 [file pone.0355856.s002.docx]

# **S2 Table.** **The median concentrations of pollutants by age group according to survival status and year of diagnosis (1999–2009 or 2010–2020).**

| **Pollutant Concentration**  **(Median (IQR))** | **Total** | **Survival Status** | | | | | | | | | | | |
| --- | --- | --- | --- | --- | --- | --- | --- | --- | --- | --- | --- | --- | --- |
|  |  | **All Age Groups** | | | **Pediatric** | | | **AYA** | | | **Adult** | | |
|  |  | **Survived** | **Died** | ***p*** | **Survived** | **Died** | ***p*** | **Survived** | **Died** | ***p*** | **Survived** | **Died** | ***p*** |
| *N* | 531 | 254 | 277 |  | 206 | 105 |  | 40 | 91 |  | 8 | 81 |  |
| No. of records | 2,538 | 1,834 | 704 |  | 1,624 | 356 |  | 169 | 209 |  | 41 | 139 |  |
| PM_2.5_ (µg/m^3^) | 35.8  (31.9-39.3) | 35.2 (31.2-39.0) | 36.8 (32.9-40.7) | <0.001 | 35.4 (31.3-39.1) | 37.0 (33.0-41.7) | <0.001 | 33.6 (29.4-38.1) | 36.3 (32.7-40.0) | 0.001 | 34.6 (32.3-38.1) | 36.8 (33.4-39.0) | 0.106 |
| PM_10_ (µg/m^3^) | 49.8  (44.5-54.8) | 49.0  (43.4-54.4) | 51.2 (46.0-56.6) | <0.001 | 49.3 (43.6-54.5) | 51.6 (46.0-57.7) | <0.001 | 47.1 (41.2-53.1) | 50.5 (45.7-55.7) | 0.001 | 48.5 (44.9-53.1) | 51.2 (46.5-54.5) | 0.114 |
| NO_2_ (ppb) | 7.2  (5.4-8.7) | 7.3 (5.3-8.7) | 7.1 (5.5-8.8) | 0.227 | 7.1 (5.3-8.5) | 6.4 (5.3-8.3) | 0.118 | 8.1 (5.6-8.8) | 7.8 (5.6-9.4) | 0.430 | 8.8 (7.6-9.3) | 8.2 (5.4-9.0) | 0.042 |
| O_3_ (ppb) | 36.4  (34.9-38.0) | 36.3 (34.8-37.9) | 36.5 (35.0-38.3) | 0.007 | 36.4 (34.9-37.9) | 36.8 (35.3-38.7) | <0.001 | 36.3 (34.7-38.4) | 36.4 (35.0-37.6) | 0.520 | 36.1 (34.7-37.1) | 35.9 (34.9-38.3) | 0.248 |
| CO (ppb) | 378.9  (338.4-411.8) | 372.2 (335.1-409.2) | 388.7 (353.6-420.7) | <0.001 | 374.0 (336.6-411.3) | 390.2 (354.4-427.0) | <0.001 | 356.4 (315.6-392.2) | 389.2 (345.0-416.3) | <0.001 | 366.2 (338.3-392.2) | 386.6 (362.5-405.4) | 0.036 |
|  |  | **Year of Diagnosis** | | | | | | | | | | | |
|  |  | **All Age Groups** | | | **Pediatric** | | | **AYA** | | | **Adult** | | |
|  |  | **1999–2009** | **2010–2020** | ***p*** | **1999–2009** | **2010–2020** | ***p*** | **1999–2009** | **2010–2020** | ***p*** | **1999–2009** | **2010–2020** | ***p*** |
| *N* | 531 | 264 | 267 |  | 165 | 146 |  | 63 | 68 |  | 36 | 53 |  |
| No. of records | 2,538 | 1,604 | 934 |  | 1,335 | 645 |  | 190 | 188 |  | 79 | 101 |  |
| PM_2.5_ (µg/m^3^) | 35.8 (31.9-39.3) | 37.2 (33.5-41.1) | 33.2 (28.4-37.4) | <0.001 | 37.1 (33.4-41.1) | 33.0 (28.3-37.0) | <0.001 | 37.6  (34.1-42.5) | 33.2 (28.4-37.5) | <0.001 | 37.2 (34.3-40.0) | 34.4 (31.0-38.4) | 0.001 |
| PM_10_ (µg/m^3^) | 49.8 (44.5-54.8) | 51.8 (46.8-57.2) | 46.3 (39.8-51.9) | <0.001 | 51.7 (46.5-57.1) | 46.1 (39.5-51.7) | <0.001 | 52.5 (47.7-58.8) | 46.3 (39.8-52.1) | <0.001 | 52.0 (48.0-55.6) | 48.2 (43.2-53.6) | 0.001 |
| NO_2_ (ppb) | 7.2 (5.4-8.7) | 7.5 (5.6-8.9) | 6.7 (5.0-8.4) | <0.001 | 7.1 (5.5-8.7) | 6.3 (4.9-8.4) | <0.001 | 8.4 (6.5-9.5) | 7.2 (5.3-8.6) | <0.001 | 9.0 (7.1-9.9) | 7.9 (5.4-8.4) | <0.001 |
| O_3_ (ppb) | 36.4 (34.9-38.0) | 36.6 (35.1-38.2) | 35.8 (34.6-37.7) | <0.001 | 36.6 (35.1-38.3) | 35.8 (34.5-37.6) | <0.001 | 36.8 (35.4-38.3) | 35.6 (34.6-37.5) | <0.001 | 36.2 (35.1-37.1) | 35.3 (34.6-38.3) | 0.309 |
| CO (ppb) | 378.9 (338.4-411.8) | 391.4 (359.9-423.9) | 345.0 (311.1-386.6) | <0.001 | 389.6 (357.3-423.9) | 343.5 (309.9-386.3) | <0.001 | 398.9 (367.5-425.2) | 345.0 (309.9-386.5) | <0.001 | 395.4 (374.6-413.3) | 367.0 (338.1-388.9) | <0.001 |

AYA, adolescent and young adult; IQR, interquartile range; *N*, number of participants; PM_2.5_, particulate matter < 2.5 μm; PM_10_, particulate matter < 10 μm; NO_2_, nitrogen dioxide; O_3_, ozone; CO, carbon monoxide.

**S2 Table** provides a comparison of median pollutant concentrations, differentiating between survival status and year of diagnosis. The findings indicate that PM_2.5_, PM_10_, and CO levels were significantly elevated among deceased patients across pediatric and AYA patients. Conversely, concentrations of all pollutants were significantly lower in those who were diagnosed between 2010-2020 compared to those diagnosed in the earlier period across all age groups, with the exception of O_3_ concentration in the adult group.
