# Supplementary material for: Air pollution and mortality in acute lymphoblastic leukemia patients across different age groups in Northern Thailand (1999–2020)
Source: PLoS One. 2026 Aug 13;21(8):e0355856. doi: 10.1371/journal.pone.0355856 (PMC13472421; doi:10.1371/journal.pone.0355856)
Supplement: Supplementary file 3 [file pone.0355856.s003.docx]

**S3 Table** provides the univariable and multivariable Cox proportional hazards model results for pediatric patients. These results suggest that pediatric patients diagnosed before 2010 exhibited a 1.7-fold increased risk of mortality. Furthermore, high PM_2.5_ levels (≥50 µg/m³) showed a tendency towards association with a higher risk of death.

# **S3 Table. Risk factors associated with death in the pediatric group with acute lymphoblastic leukemia.**

| **Factor** | **Univariable Analysis** | | | **Multivariable Analysis** | | |
| --- | --- | --- | --- | --- | --- | --- |
|  | **HR** | **95% CI** | ***p*** | **aHR** | **95% CI** | ***p*** |
| **At diagnosis** |  |  |  |  |  |  |
| Age (years old) |  |  |  |  |  |  |
| - < 1 | 2.42 | 1.29–4.53 | 0.01 | 2.05 | 0.93–4.51 | 0.07 |
| - 1–9 | Ref | – | – | – | – | – |
| - 10–14 | 1.18 | 0.75–1.84 | 0.48 | 1.39 | 0.84–2.29 | 0.20 |
| Male | 1.15 | 0.81–1.65 | 0.43 | – | – | – |
| Hill tribe ethnicity | 0.89 | 0.53–1.51 | 0.67 | – | – | – |
| Year of diagnosis |  |  |  |  |  |  |
| - 1999–2009 | 1.82 | 1.24–2.67 | 0.002 | 1.72 | 1.06–2.80 | 0.03 |
| - 2010–2020 | Ref | – | – | – | – | – |
| Family history of cancer | 1.05 | 0.43–2.56 | 0.92 | – | – | – |
| Maternal smoking | 0.70 | 0.34–1.43 | 0.32 | – | – | – |
| **Time-updated variables** |  |  |  |  |  |  |
| PM_2.5_ concentration ≥ 50 µg/m^3^ | 2.02 | 1.10–3.70 | 0.02 | 1.92 | 0.96–3.83 | 0.06 |
| PM_10_ concentration ≥ 55 µg/m^3^ | 1.16 | 0.76–1.78 | 0.49 | – | – | – |
| NO_2_ concentration ≥ 8.5 ppb | 0.94 | 0.59–1.50 | 0.79 | – | – | – |
| O_3_ concentration ≥ 37.8 ppb | 0.93 | 0.59–1.48 | 0.77 | – | – | – |
| CO concentration ≥ 410 ppb | 1.33 | 0.87–2.01 | 0.18 | 0.92 | 0.56–1.53 | 0.76 |

HR, hazard ratio; aHR, adjusted HR; CI, confidence interval; PM_2.5_, particulate matter < 2.5 μm; PM_10_, particulate matter < 10 μm; NO_2_, nitrogen dioxide; O_3_, ozone; CO, carbon monoxide.
