# Supplementary material for: Air pollution and mortality in acute lymphoblastic leukemia patients across different age groups in Northern Thailand (1999–2020)
Source: PLoS One. 2026 Aug 13;21(8):e0355856. doi: 10.1371/journal.pone.0355856 (PMC13472421; doi:10.1371/journal.pone.0355856)
Supplement: Supplementary file 4 [file pone.0355856.s004.docx]

**S4 Table** provides the univariable and multivariable Cox proportional hazards model results for adolescent and young adult patients. The findings indicate that patients aged 30 years or older had a 1.7-fold increased risk of mortality. NO_2_ levels of 8.5 ppb or higher showed a tendency towards association with a higher risk of death.

# **S4 Table.** **Risk factors associated with death in the adolescent and young adult group with acute lymphoblastic leukemia.**

| **Factors** | **Univariable Analysis** | | | **Multivariable Analysis** | | |
| --- | --- | --- | --- | --- | --- | --- |
|  | **HR** | **95% CI** | ***p*** | **aHR** | **95% CI** | ***p*** |
| **At diagnosis** |  |  |  |  |  |  |
| Age (years old) |  |  |  |  |  |  |
| - 15–19 | Ref | – | – | – | – | – |
| - 20–29 | 1.18 | 0.74–1.87 | 0.49 | 0.92 | 0.54–1.56 | 0.76 |
| - 30–39 | 1.97 | 1.24–3.15 | 0.004 | 1.69 | 1.01–2.85 | 0.047 |
| Male | 0.83 | 0.57–1.22 | 0.34 | – | – | – |
| Hill tribe ethnicity | 1.38 | 0.78–2.42 | 0.27 | – | – | – |
| Year of diagnosis |  |  |  |  |  |  |
| - 1999–2009 | 1.48 | 1.00–2.19 | 0.05 | 1.34 | 0.85–2.12 | 0.21 |
| - 2010–2020 | Ref | – | – | – | – | – |
| Family history of cancer | 0.93 | 0.41–2.12 | 0.86 | – | – | – |
| Smoking status | 0.95 | 0.56–1.62 | 0.85 | – | – | – |
| **Time-updated variables** |  |  |  |  |  |  |
| PM_2.5_ concentration ≥ 50 µg/m^3^ | 1.39 | 0.56–3.46 | 0.47 | – | – | – |
| PM_10_ concentration ≥ 55 µg/m^3^ | 1.30 | 0.81–2.09 | 0.28 | – | – | – |
| NO_2_ concentration ≥ 8.5 ppb | 0.64 | 0.39–1.05 | 0.08 | 0.60 | 0.36–1.01 | 0.051 |
| O_3_ concentration ≥ 37.8 ppb | 0.73 | 0.42–1.24 | 0.24 | 0.67 | 0.38–1.17 | 0.16 |
| CO concentration ≥ 410 ppb | 1.36 | 0.85–2.17 | 0.20 | 1.33 | 0.81–2.18 | 0.27 |

HR, hazard ratio; aHR, adjusted HR; CI, confidence interval; PM_2.5_, particulate matter < 2.5 μm; PM_10_, particulate matter < 10 μm; NO_2_, nitrogen dioxide; O_3_, ozone; CO, carbon monoxide.
