# Supplementary material for: Air pollution and mortality in acute lymphoblastic leukemia patients across different age groups in Northern Thailand (1999–2020)
Source: PLoS One. 2026 Aug 13;21(8):e0355856. doi: 10.1371/journal.pone.0355856 (PMC13472421; doi:10.1371/journal.pone.0355856)
Supplement: Supplementary file 5 [file pone.0355856.s005.docx]

**S5 Table** presents the univariable and multivariable Cox proportional hazards model results for adult patients. The results suggest that adults aged 60 years or older had a 1·4-fold increased risk of mortality. None of the studied air pollutants demonstrated an association with mortality risk.

# **S5 Table.** **Risk factors associated with death in the adult group with acute lymphoblastic leukemia.**

| **Factors** | **Univariable Analysis** | | | **Multivariable Analysis** | | |
| --- | --- | --- | --- | --- | --- | --- |
|  | **HR** | **95% CI** | ***p*** | **aHR** | **95% CI** | ***p*** |
| **At diagnosis** |  |  |  |  |  |  |
| Age (years) |  |  |  |  |  |  |
| - 40–49 | Ref | – | – | – | – | – |
| - 50–59 | 1·43 | 0·85–2·41 | 0·17 | 1.42 | 0.83–2.42 | 0.20 |
| - ≥ 60 | 2·12 | 1·32–3·41 | 0·002 | 1.97 | 1.21–3.19 | 0.01 |
| Male | 1·40 | 0·94–2·10 | 0·10 | 1.40 | 0.93–2.10 | 0.10 |
| Hill tribe ethnicity | 0·98 | 0·36–2·68 | 0·97 | – | – | – |
| Year of diagnosis |  |  |  |  |  |  |
| - 1999–2009 | 1·52 | 1·02–2·27 | 0·04 | 1.48 | 0.98–2.21 | 0.06 |
| - 2010–2020 | Ref | – | – | – | – | – |
| Family history of cancer | 0·46 | 0·19–1·14 | 0·09 | 0.63 | 0.24–1.62 | 0.33 |
| Smoking status | 0·91 | 0·53–1·59 | 0·75 |  |  |  |
| **Time-updated variables** |  |  |  |  |  |  |
| PM_2·5_ concentration ≥ 50 µg/m^3^ | 0·84 | 0·36–1·94 | 0·68 | – | – | – |
| PM_10_ concentration ≥ 55 µg/m^3^ | 1·01 | 0·60–1·71 | 0·97 | – | – | – |
| NO_2_ concentration ≥ 8·5 ppb | 0·87 | 0·54–1·40 | 0·56 | – | – | – |
| O_3_ concentration ≥ 37·8 ppb | 0·96 | 0·59–1·56 | 0·87 | – | – | – |
| CO concentration ≥ 410 ppb | 0·86 | 0·51–1·46 | 0·57 | – | – | – |

HR, hazard ratio; aHR, adjusted HR; CI, confidence interval; PM_2·5_, particulate matter < 2·5 μm; PM_10_, particulate matter < 10 μm; NO_2_, nitrogen dioxide; O_3_, ozone; CO, carbon monoxide.
