# Supplementary material for: Air pollution and mortality in acute lymphoblastic leukemia patients across different age groups in Northern Thailand (1999–2020)
Source: PLoS One. 2026 Aug 13;21(8):e0355856. doi: 10.1371/journal.pone.0355856 (PMC13472421; doi:10.1371/journal.pone.0355856)
Supplement: Supplementary file 6 [file pone.0355856.s006.docx]

**S1 Fig.** illustrates the pattern of average yearly concentrations of the pollutants studied (PM_2·5_, PM_10_, NO_2_, O_3_, and CO) from 2003 to 2020. All pollutants, with the exception of O_3_, exhibited annual fluctuations in their levels.

**S1 Fig. Average annual concentrations of (a) PM_2·5_ (particulate matter < 2.5 μm), (b) PM_10_ (particulate matter < 10 μm), (c) NO_2_ (nitrogen dioxide), (d) O_3_ (ozone), and (e) CO (carbon monoxide).**
