# Supplementary material for: Air pollution and mortality in acute lymphoblastic leukemia patients across different age groups in Northern Thailand (1999–2020)
Source: PLoS One. 2026 Aug 13;21(8):e0355856. doi: 10.1371/journal.pone.0355856 (PMC13472421; doi:10.1371/journal.pone.0355856)
Supplement: Supplementary file 7 [file pone.0355856.s007.docx]

**S2 Fig.** illustrates the distribution and comparison of each pollutant across age groups and years of diagnosis. Only NO_2_ showed variation between age groups. Similarly, all pollutants, except O_3_ in adult patients, showed differences across years of diagnosis within each age group.

# **S2 Fig. The median concentrations of (a) PM_2.5_ by age group, (b) PM_2.5_ by age group and year of diagnosis, (c) PM_10_ by age group, (d) PM_10_ by age group and year of diagnosis, (e) NO_25_ by age group, (f) NO_2_ by age group and year of diagnosis, (g) O_3_ by age group, (h) O_3_ by age group and year of diagnosis, (i) CO by age group, (j) CO by age group and year of diagnosis during the diagnosis of ALL to death or last visit.** SD, standard deviation; PM_2·5_, particulate matter < 2·5 μm; PM_10_, particulate matter < 10 μm; NO_2_, nitrogen dioxide; O_3_, ozone; CO, carbon monoxide.
